# Synthetic Peptides as Protein Mimics

**DOI:** 10.3389/fbioe.2015.00211

**Published:** 2016-01-19

**Authors:** Andrea Groß, Chie Hashimoto, Heinrich Sticht, Jutta Eichler

**Affiliations:** ^1^Department of Chemistry and Pharmacy, University of Erlangen-Nuremberg, Erlangen, Germany; ^2^Institute of Biochemistry, University of Erlangen-Nuremberg, Erlangen, Germany

**Keywords:** protein–protein interactions, protein mimics, peptides, structure-based design, biomaterials

## Abstract

The design and generation of molecules capable of mimicking the binding and/or functional sites of proteins represents a promising strategy for the exploration and modulation of protein function through controlled interference with the underlying molecular interactions. Synthetic peptides have proven an excellent type of molecule for the mimicry of protein sites because such peptides can be generated as exact copies of protein fragments, as well as in diverse chemical modifications, which includes the incorporation of a large range of non-proteinogenic amino acids as well as the modification of the peptide backbone. Apart from extending the chemical and structural diversity presented by peptides, such modifications also increase the proteolytic stability of the molecules, enhancing their utility for biological applications. This article reviews recent advances by this and other laboratories in the use of synthetic protein mimics to modulate protein function, as well as to provide building blocks for synthetic biology.

## Introduction

The detailed insight into the human genome does not in itself enable a comprehensive understanding of human protein function, health, and disease. In the post-genome era, an important challenge is the structural and functional analysis of the gene products, i.e., proteins. Proteins play a major role in almost all biological processes, including enzymatic reactions, structural integrity of cells, organs and tissues, cell motility, immune responses, signal transduction, and sensing. All protein-mediated biological processes are based on specific interactions between proteins and their ligands. Therefore, exploring disease-associated protein–ligand and protein–protein interactions is essential to gain insight into the molecular mechanisms underlying diseases and other phenomena, as well as for the development of novel therapeutic strategies.

Molecules that present the binding sites of proteins, which are involved in a disease-associated protein–protein interaction, are promising candidates for therapeutic intervention. Such binding site mimetic molecules can be generated either through recombinant protein synthesis or by means of chemical peptide synthesis. A specific advantage of synthetic peptides is that they can be generated as exact copies of protein fragments as well as in diverse chemical modifications, which include the incorporation of a large range of non-proteinogenic amino acids, as well as the modification of the peptide backbone. Apart from extending the chemical and structural diversity presented by peptides, such modifications also increase the proteolytic stability of the molecules, enhancing their potential as drug candidates.

**Figure 1:**
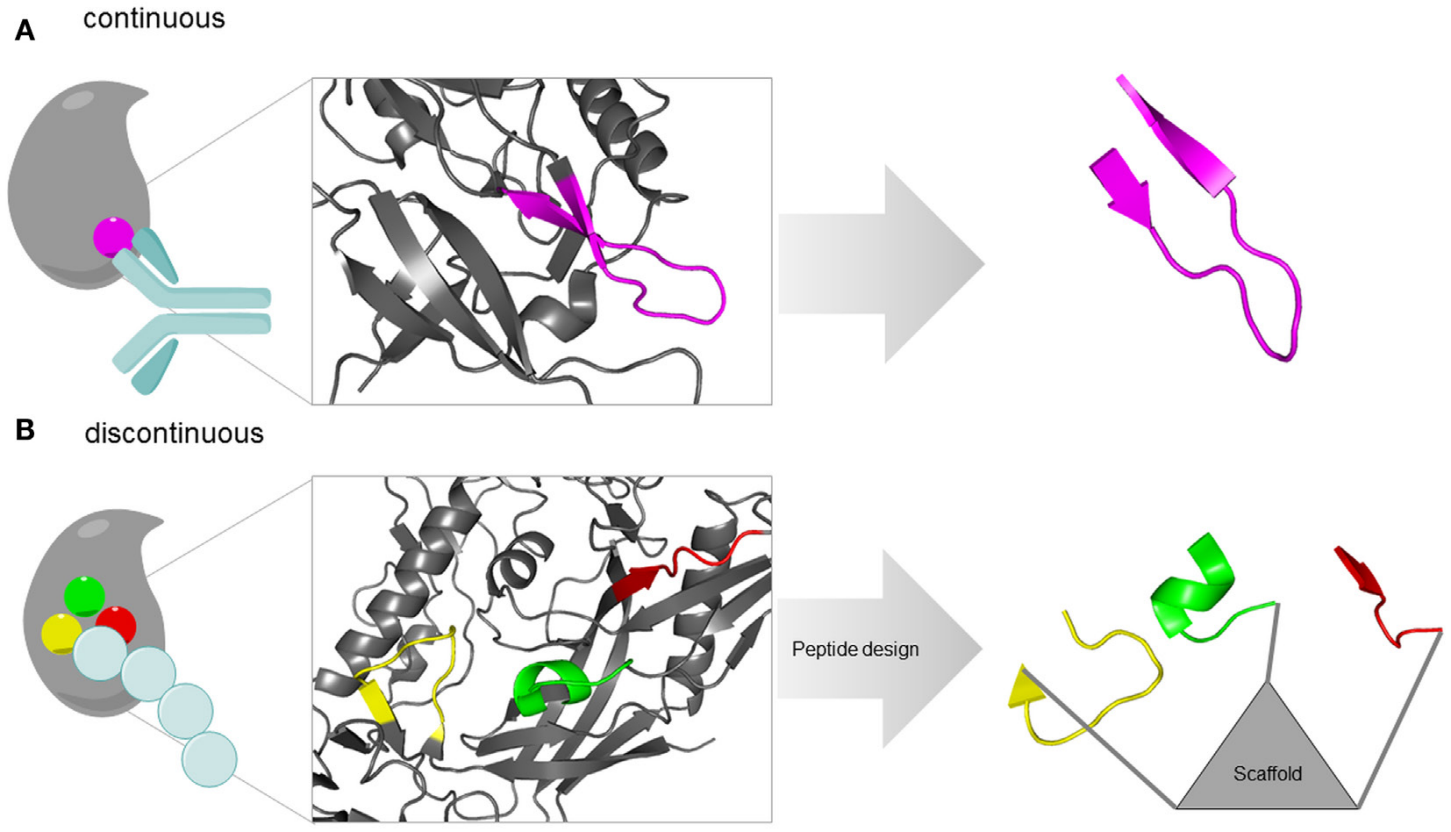
**Types of protein-binding sites illustrated by the HIV-1 envelope protein gp120**. **(A)** Continuous epitope of gp120 for an antibody [pdb 4TVP (Pancera et al., 2014)]. The epitope (V3-loop tip, pink) is located in a single sequence stretch and can be reproduced in a single peptide. **(B)** Discontinuous protein-binding site of gp120 for its receptor CD4 [pdb 4TVP (Pancera et al., 2014)]. The binding site is located in three sequentially discontinuous segments of the protein sequence (yellow, green, and red). In a mimetic peptides, these three fragments are presented through a molecular scaffold.

Three conceptually different approaches are available for the design of protein-binding site mimetic peptides. These approaches are based on one or more of the following information about the proteins of interest: structure, sequence, and function. In random combinatorial methods that are based solely on protein function, such as phage display (Li and Caberoy, 2010) and synthetic peptide combinatorial libraries (Houghten et al., 1999), respectively, large populations of peptides are screened for binders to the respective partner protein, or for inhibitors of the protein–protein interaction of interest. A strategy termed peptide scanning is based on the synthesis of the entire protein sequence – or large parts of it – in the shape of short, overlapping peptides, which are then individually tested for binding to the respective partner protein (Frank, 2002), enabling the identification of protein-binding sites. The utility of this method, however, is largely limited to the identification of sequentially continuous binding sites, which are located in a protein sequence stretch of consecutive amino acids (Figure 1A). Structure-based design, finally, involves the design and generation of protein-binding site mimics based on the 3D structure of the protein–protein complexes (Eichler, 2008). This structural information enables the design and generation of mimics of continuous, as well as of sequentially discontinuous protein-binding sites, which are composed of two or more protein segments that are distant in protein sequence, but brought into spatial proximity through protein folding (Figure 1B). Mimicking such discontinuous protein-binding sites by synthetic peptides typically involves presentation of the respective protein fragments through a molecular scaffold (Figure 1B).

Here, we review strategies for the use of synthetic peptides as protein mimics. Focusing on structure-based design, the potential of such peptides as drugs against diseases, such as viral and bacterial infections, cancer, as well as autoimmune diseases, are discussed.

## Toolbox for Peptide Synthesis: Non-Proteinogenic Amino Acids and Site-Selective Ligation

**Figure 2:**
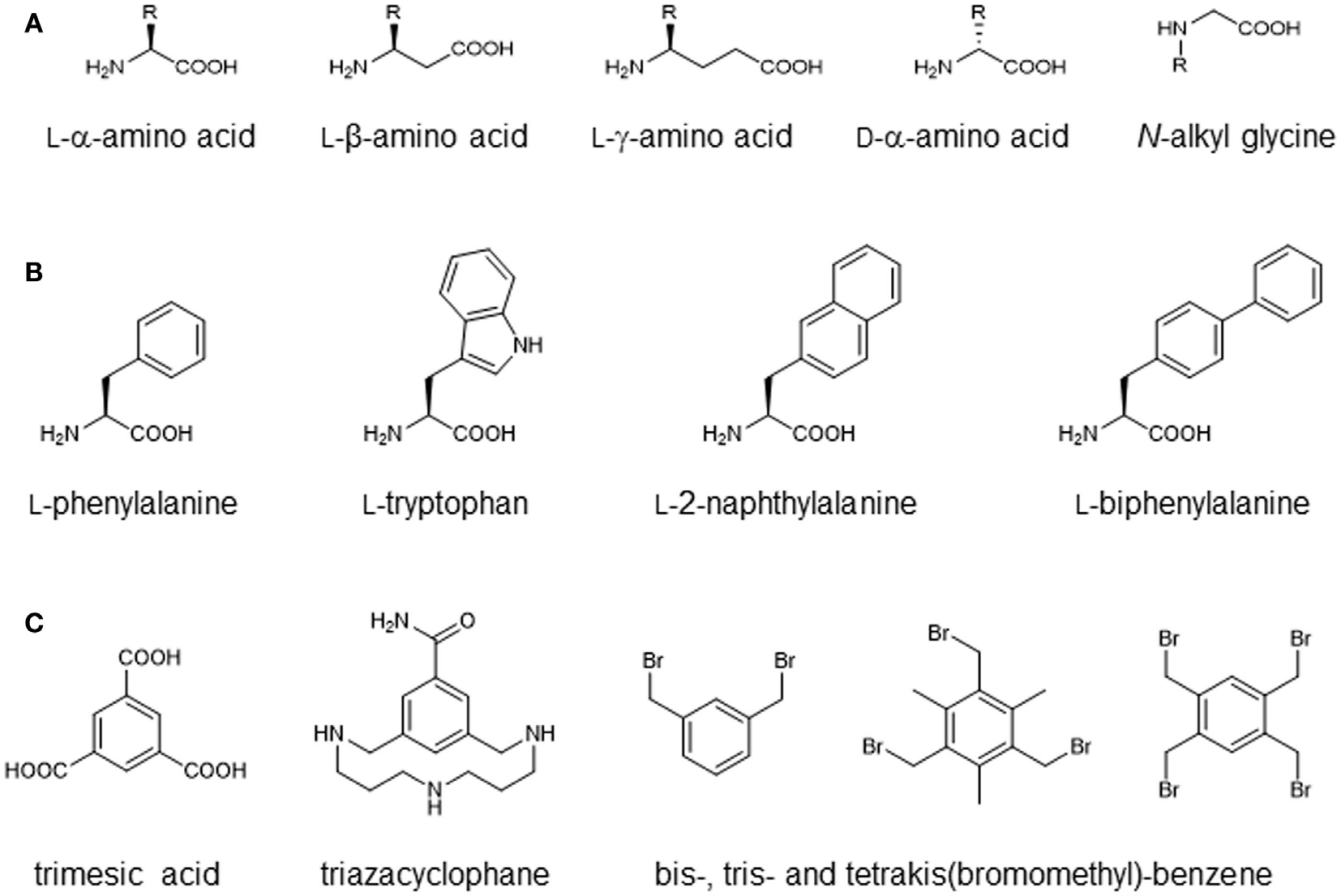
**Building blocks for chemical peptide synthesis**. **(A)** Amino acid derivatives with modified backbone length and side-chain orientation. **(B)** Amino acid derivatives with modified aromatic side chains. **(C)** Scaffolds for multivalent or discontinuous peptide presentation.

Most current methods for the chemical synthesis of peptides utilize Merrifield’s concept of solid-phase synthesis (Merrifield, 1963), which enables the synthesis of peptides and small proteins of up to 100 amino acids. A major advantage of chemical peptide synthesis, as compared to recombinant protein synthesis, is the extended set of amino acids and other building blocks that can be incorporated, which includes d-amino acids, as well as a wide range of non-proteinogenic amino acids (Figure 2). While the recombinant synthesis of proteins containing non-proteinogenic amino acids is possible only through alternative codon usage (Mehl et al., 2003), hundreds of such building blocks are commercially available for the use in chemical peptide synthesis. This opens the door to improved biological activity and peptide stability, as well as structural modifications. One possibility is the use of β- and γ-amino acids (Seebach et al., 2004), which differ from α-amino acids in having one or two additional methylene groups between the carboxy and the amino function of the amino acid (Figure 2A). Peptides composed of these amino acids are stable against proteolysis *in vitro* and *in vivo*, as well as metabolism and degradation by microbial colonies (Seebach et al., 2004; Seebach and Gardiner, 2008). On the functional side, they can act similar to the natural α-peptides, as examplified by β- and γ-peptide agonists of naturally occurring α-peptide hormones such as somatostatin (Seebach et al., 2004; Seebach and Gardiner, 2008).

Another possibility is the use of d-amino acids. Due to the chirality of the Cα-atom, amino acids exist in two different stereoisomers (l and d). While recombinantly synthesized peptides and proteins are typically composed entirely of l-amino acids, chemical peptide synthesis can also use d-amino acids, which has been shown to increase the proteolytic stability while maintaining biolocical activity when d-amino acids are introduced at defined positions of an antimicrobial peptide (Hong et al., 1999b). At other positions, on the other hand, using d-amino acids instead of l-amino acids had the opposite effect due to structural damage to the peptide (Hong et al., 1999b).

Furthermore, oligomers of *N*-alkyl glycine monomers, termed peptoids, have been introduced as proteolytically stable peptide derivatives (Simon et al., 1992) (Figure 2A). As the amide hydrogen is missing in peptoids, the typical backbone hydrogen bonds present in proteins and peptides cannot be formed, altering the conformational preferences of these molecules. Peptoids have been used as mimics of antimicrobial peptides (termed as ampetoids) (Chongsiriwatana et al., 2008; Mojsoska et al., 2015) as well as novel therapeutics (Zuckermann and Kodadek, 2009).

In addition to alteration of the peptide backbone, the use of non-proteinogenic amino acids enables the introduction of chemical moieties that are not presented by the proteinogenic amino acids, and which can be used to dissect the binding mode of peptides. A prominent example is the substitution of aromatic side chains of phenylalanine or tryptophan with larger aromatic groups such as naphthyl or biphenyl (Figure 2B), which increases the size and hydrophobicity of the side chain and affects π-stacking with the respective protein ligand (Muraki et al., 2000; Bachmann et al., 2011). Functionalized and orthogonally protected amino acids are often used for chemo selective ligation strategies (Tornoe et al., 2002; Kimmerlin and Seebach, 2005). In addition, lysine, among other amino acids, can be used for the synthesis of branched peptides (Franke et al., 2007). Furthermore, a range of scaffold molecules, such as trimesic acid derivatives (Berthelmann et al., 2014), triazacyclophane derivatives (Opatz and Liskamp, 2001; Chamorro et al., 2009), bis-, tris-, and tetra-kis(bromomethyl)-benzene (CLIPS technology) (Timmerman et al., 2005) (Figure 2C), as well as cyclic β-tripeptide derivatives (Seebach and Gardiner, 2008) have been introduced for the generation of multivalent peptides.

## Protein Secondary Structure Mimics

The three-dimensional (3D) arrangement of proteins contains unstructured, as well as structured regions, in which peptide chains are organized into secondary structures, such as α-helices and β-sheets. As α-helices and β-sheets mediate protein folding and protein–protein interactions, they are related to various biochemical phenomena and diseases (Fairlie et al., 1998). These secondary structures are stabilized by hydrogen bonds between amide nitrogen and carbonyl oxygen atoms. Bullock et al. have analyzed the full set of helical protein interfaces in the Protein Data Bank (Berman et al., 2000) and found that about 62% of the helical interfaces contribute to protein–protein interactions (Bullock et al., 2011). Although natural proteins contain less β-sheet structure than α-helical structure, β-sheets contribute to protein aggregation, as well as to protein–protein interactions. Thus, peptides that mimic α-helices and β-sheets of proteins are attractive targets for drug development and tools to explore protein binding mechanism. A range of α-helix and β-sheet mimics have been developed, which will be discussed below. The various strategies of mimicking protein-binding sites through secondary structure mimics have also been extensively reviewed recently (Pelay-Gimeno et al., 2015).

### α-Helix Mimics

The α-helical conformation of a peptide can be stabilized, and even induced, by introducing covalent links between amino acid side chains at selected positions. These links can be formed by lactam (Ösapay and Taylor, 1992; Yu and Taylor, 1999; Sia et al., 2002; Yang et al., 2004; Mills et al., 2006) and disulfide bridges (Jackson et al., 1991; Leduc et al., 2003), triazole-based linkages (Scrima et al., 2010; Kawamoto et al., 2011; Madden et al., 2011), and hydrocarbon staples (Blackwell and Grubbs, 1998; Schafmeister et al., 2000; LaBelle et al., 2012; Verdine and Hilinski, 2012; Brown et al., 2013; Chang et al., 2013; Nomura et al., 2013; Walensky and Bird, 2014; Chu et al., 2015). Replacing hydrogen bonds by salt bridges has been reported by Otaka et al. as an alternative means of stabilizing α-helices (Otaka et al., 2002). Further examples for hydrogen bond surrogates include cation–π interaction (Olson et al., 2001; Shi et al., 2002; Tsou et al., 2002) and π–π interaction (Albert and Hamilton, 1995).

Foldamers are a very prominent class of α-helix mimetic peptides. They are composed of β-amino acid (Seebach and Matthews, 1997; Gellman, 1998; Cheng et al., 2001; Martinek and Fulop, 2003), α/β-amino acid oligomers (Johnson and Gellman, 2013), or *N*-substituted glycine residues (peptoids) (Sun and Zuckermann, 2013). Such foldamers have been shown to inhibit the proteolytic activity of γ-secretase (Imamura et al., 2009), an enzyme that is involved in the processing of amyloid-β (Aβ) in Alzheimer’s disease, by blocking the initial substrate binding site of γ-secretase (Lichtenthaler et al., 1999). For these foldamers, the conformationally constrained β-amino acid *trans*-2-aminocyclopentanecarboxylic acid (ACPC) was used as a building block. As such α-helix mimics can increase α-helicity, stability, and cell-permeability, they are increasingly attracting the attention both in academia and the pharmaceutical industry as candidates for novel therapeutics. Apart from biomedical use, α-helical peptide mimics are also of interest as biomaterials, such as self-assembling nanotubes (Burgess et al., 2015) and hydrogels (Mehrban et al., 2015).

### β-Sheet Mimics

In β-sheets, two or more β-strands are connected via loops or turns, and the parallel or antiparallel orientation of β-strands is stabilized by hydrogen bonds between carbonyl oxygen atoms in one strand and amide nitrogen atoms of the opposite strand. Methods to mimic turn structures include macrocyclization as well as the use of turn-inducing building blocks, such as a dipeptide of d-proline and l-proline (Robinson, 2008), or α-aminoisobutyric acid in combination with either a d-α-amino acid or an achiral α-amino acid (Aravinda et al., 2002; Masterson et al., 2007). One noteworthy example for macrocyclization used cyclic cysteine ladders of θ-defensin as a scaffold to stabilize a turn structure (Conibear et al., 2014). The cyclic cysteine ladder of θ-defensin comprises two antiparallel β-strands connected via two β-turns, and has a high thermal and serum stability. Grafting of the integrin-binding peptide Arg–Gly–Asp (RGD) onto this molecule resulted in 10-fold increase in affinity to integrin, illustrating the utility of θ-defensin as a molecular scaffold.

It has been difficult to develop robust chemical models of β-sheets, which tolerate a wide range of amino acid sequences because amyloidogenic sequences vary enormously and folding of β-sheet mimics depends on their amino acid sequences. Woods et al. overcame this problem by using 42-membered rings, which contain two strands connected via two δ-linked ornithine turns (Woods et al., 2007). Forty-two-membered ring macrocyclic β-sheets present a pentapeptide β-strand on one side (recognition strand), while the other β-strand contains the unnatural amino acid Hao (5-hydrazino-2-methoxybenzoic acid) and two α-amino acids. The relatively rigid structure of Hao-containing peptides preserves the structure of the recognition strand, and at the time serves as a template for the recognition strand. Furthermore, Hao is useful for the intermolecular β-sheet interaction to form fibril-like assembled oligomers (Pham et al., 2014). Similar to α-helical peptides, β-sheet mimics have also been used for biomaterials, such as nanotubes (Hamley, 2014).

## Stimuli Responsive Peptides in Biomaterial Engineering

Some peptides are able to be structurally rearranged in response to external stimuli, such as temperature, pH, ionic strength, and presence of special ions and light. In 2006, Mart et al. (2006) reviewed different responsive systems based on peptides and their applications, including switchable surfaces, nanoparticle (dis)-assembly, hydrogel-formation, metal ion sensing, and electron transfer. In addition, special applications in medicine, such as drug delivery, tissue engineering, tissue regeneration, wound healing, and nerve cell regrowth rely upon stimuli-responsive peptides. Several conformational transitions of peptides have been reported, ranging from α-helix to random coil and *vice versa* or β-sheet to random coil and *vice versa*, among others. In this review, two selected examples are presented.

**Figure 3:**
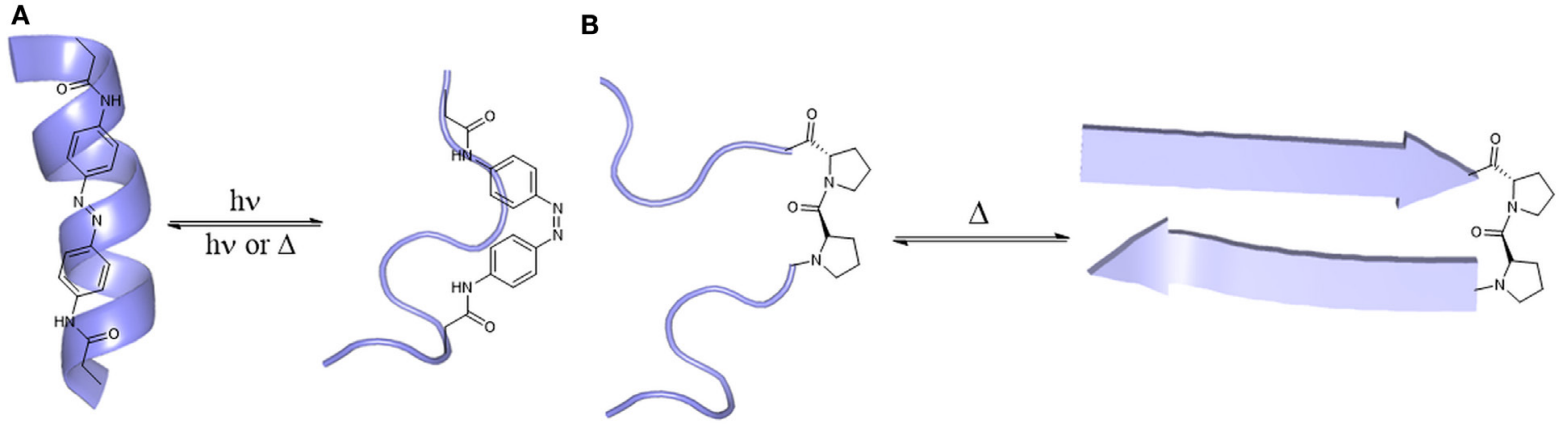
**Stimuli responsive peptides**. **(A)** Transition of azobenzene (*trans*/*cis*)-derivatized helical peptides upon to light stimulus (Beharry and Woolley, 2011). **(B)** Transition of a random coil peptide upon temperature stimulus (Pochan et al., 2003).

One example is the use of an azobenzene moiety as light-sensitive switch (Woolley, 2005; Renner and Moroder, 2006). As a photoswitchable device, azobenzene, which is more stable in the *trans*-conformation, can switch into *cis*-conformation upon irradiation with light at 340 nm, leading to a 3.5 Å shortening of the C–C-distance of azobenzene (Fliegl et al., 2003; Beharry and Woolley, 2011). Incorporation of the reactive azobenzene derivative 3,3′-bis(sulfonate)-4,4′-bis(chloroacetamido)azobenzene at defined positions of the sequence can result either in a loss of helical conformation (positions *i*, *i* + 11, Figure 3A) or in helix stabilization (positions *i*, *i* + 7), upon light stimulus (Woolley, 2005). To make this approach more feasible for *in vivo* application, longer wavelengths should be used for azobenzene isomerization, considering UV-light scattering through cells and tissues. Samanta et al. (2013) recently reported an azobenzene derivative that can be switched using red light (630–660 nm), enabling the development of photo-switchable compounds for *in vivo* use.

Another example of stimuli-responsive peptides is the temperature-dependent formation of hydrogels by β-sheet peptides. Pochan et al. (2003) designed a β-hairpin mimic called Max3 (Figure 3B) that undergoes gelation upon heating (*T*_gel_ = 60°C), which was completely reversible while cooling. This peptide is composed of alternating non-polar and polar amino acids bridged via a type II’ β-turn. Other peptides undergo non-reversible hydrogelation when heated (Max1, Max2) (Pochan et al., 2003). These β-hairpin peptides were the starting point for the design of peptides whose folding can be triggered by UV light (Haines et al., 2005), changes in pH (Rajagopal et al., 2009), or recognition of electronegative cancer cell membranes (Sinthuvanich et al., 2012). Because of their biocompatibility, biodegradability, weak immunogenicity and selectivity, peptidic hydrogels can serve as potential cancer drugs and antimicrobials, as well as for wound healing (Mart et al., 2006; Branco et al., 2011).

## Protein Mimics in Biomedical Research

Current drug discovery and development approaches are focused on three different types of molecules (Craik et al., 2013; Fosgerau and Hoffmann, 2015). The traditional approach of using small molecules as drugs is still widely used. While small molecules have been shown to be excellent tools to block the catalytic site of enzymes, as well as the ligand binding sites of numerous receptors, they are less promising for the inhibition of protein–protein interactions, which often involve larger interfaces, which typically cannot be adequately addressed by small molecules. Therefore, protein-based drugs, so-called Biologics, are increasingly used as inhibitors of protein–protein interactions. Many proteins, however, have additional effector functions or binding sites for other ligands, causing problems in *in vivo* applications. Furthermore, proteins can be immunogenic, resulting in immunological clearance before reaching their target site. As an alternative to both small molecule and protein-based drugs, peptides are becoming more relevant as drug candidates, as documented by an increasing number of peptide drugs approved for clinical use (Fosgerau and Hoffmann, 2015). Due to their potential for highly specific binding, combined with low immunogenicity, peptides are promising candidates as inhibitors of protein–protein interactions.

Specific protein–protein interactions are involved in the pathogenesis of numerous diseases. The design and generation of peptides that mimic the respective protein-binding site, as potential inhibitors of the interactions, is therefore a promising therapeutic strategy. Such mimetic molecules are typically designed based on the 3D structure of the protein–protein complex, which yields information on the location of the binding sites within the proteins, as well as the hot spot amino acids directly involved in the intermolecular interaction (Eichler, 2008). This general strategy will be illustrated here using examples of the various protein–protein interactions, which are involved in the entry of the human immunodeficiency virus type 1 (HIV-1) into cells. Furthermore, a range of protein-mimicking peptides used in the treatment of cancer and as antibiotics or anti-inflammatory compounds, will be reviewed.

### Peptides as Mimics of the Viral Spike of HIV-1

The highly active antiretroviral therapy (HAART) has been a breakthrough in the treatment of HIV-1 infection, leading to an effective reduction of morbidity and mortality through drastic suppression of viral replication and, hence, reduction of plasma HIV-1 viral load. HAART consists of a mixture of at least three different drugs with at least two different molecular targets [for details see Arts and Hazuda (2012)]. Almost all of these drugs are small molecules that address intracellular targets. Due to the high genetic variability of HIV-1, the virus is able to rapidly become resistant against drugs. Therefore, there is an ongoing need for new therapeutic strategies against HIV-1. One of these strategies is the prevention of HIV-1 entry into its host cell by blocking the interactions between viral and host proteins that are involved in the entry process. This can be achieved by using peptides, which mimic the binding sites of the involved proteins.

Entry of HIV-1 into its host cells is initiated by a cascade of protein–protein interactions between the viral and host cell proteins. These interactions involve the trimeric viral spike, composed of glycoproteins gp120 and gp41, as well as the primary receptor CD4 and corecptors CCR5 and CXCR4 on the host cell (Wilen et al., 2012).

**Figure 4:**
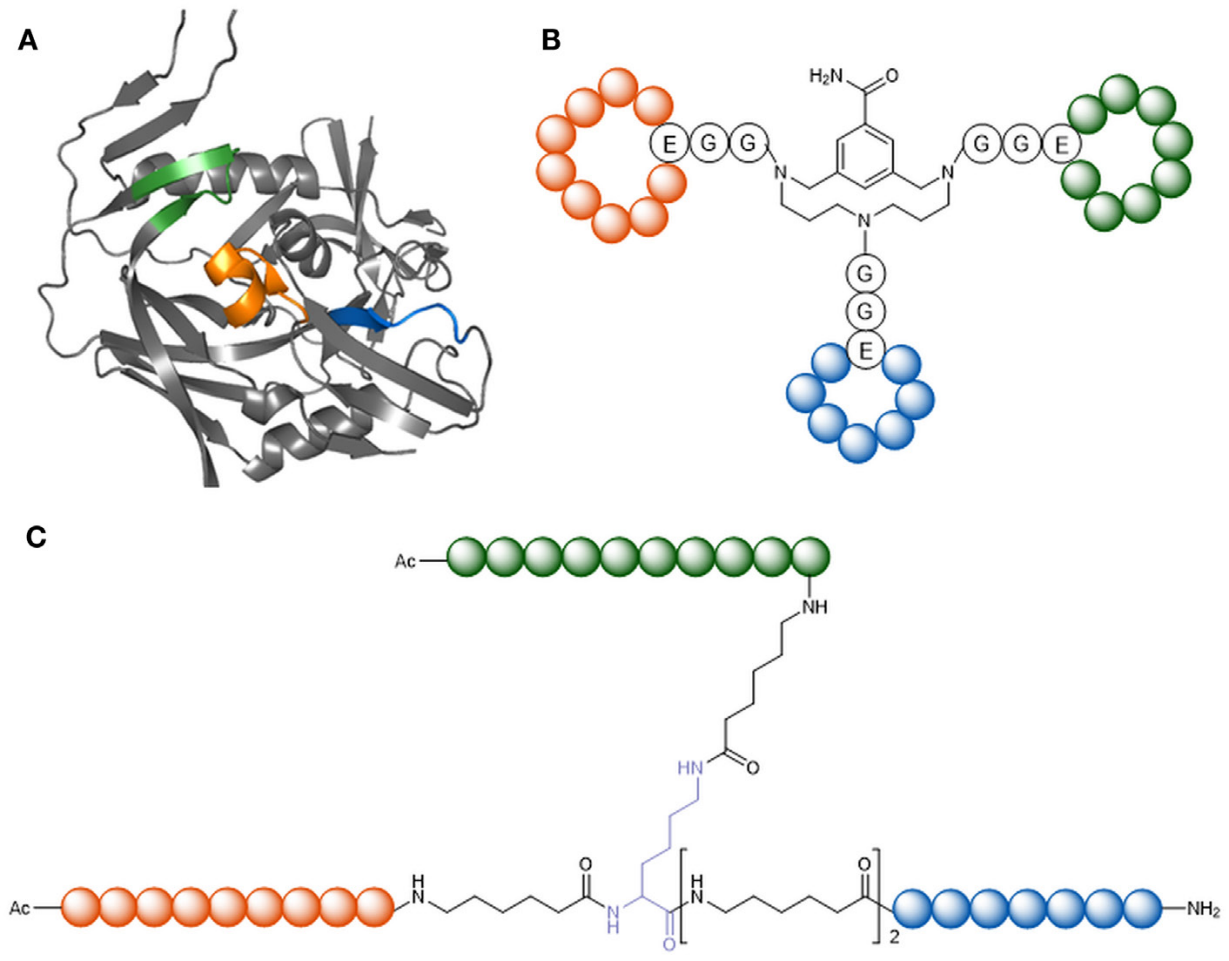
**Peptide mimics of the CD4-binding site of gp120**. **(A)** X-ray structure of gp120 in the CD4-bound conformation [1GC1 (Kwong et al., 1998)]. Highlighted in orange, blue, and green are the fragments forming the discontinuous CD4 binding site. **(B)** CD4bs-M (Franke et al., 2007). **(C)** CD4-binding site mimic with triazacyclophane scaffold (Chamorro et al., 2009).

The initial event of HIV-1 entry is an interaction of viral gp120 with the host receptor CD4. In contrast to the generally high genetic variability of HIV-1, the CD4-binding site of gp120 is highly conserved. Peptides mimicking the CD4-binding site are therefore promising candidates as HIV-1 entry inhibitors. Furthermore, as the epitopes of various broadly neutralizing anti-HIV-1 antibodies have been shown to overlap the CD4-binding site, this part of gp120 is an immunogen candidate for the generation of HIV-1 neutralizing antibodies. Based on the X-ray structure of gp120 in complex with CD4 (Kwong et al., 1998) (Figure 4A), novel peptides that mimic the CD4-binding site have been developed (Figure 4) (Franke et al., 2007; Chamorro et al., 2009). A special characteristic of these peptides is the fact that they present three sequentially discontinuous fragments of the gp120 sequence, either in linear form, or as cyclic loops, on molecular scaffolds, such as a branched peptide composed of spacer amino acids, CD4bs-M (Figure 4A), and a triazacyclophane scaffold (Figure 4B). While the triazacyclophane scaffold peptide did not affect HIV-1 infection (Chamorro et al., 2009), CD4bs-M was surprisingly found to strongly enhance HIV-1 infection of both CD4 positive and CD4 negative cells, and this effect could be linked to a strong tendency of the peptide to assemble into amyloid fibrils (Groß et al., 2015b).

**Figure 5:**
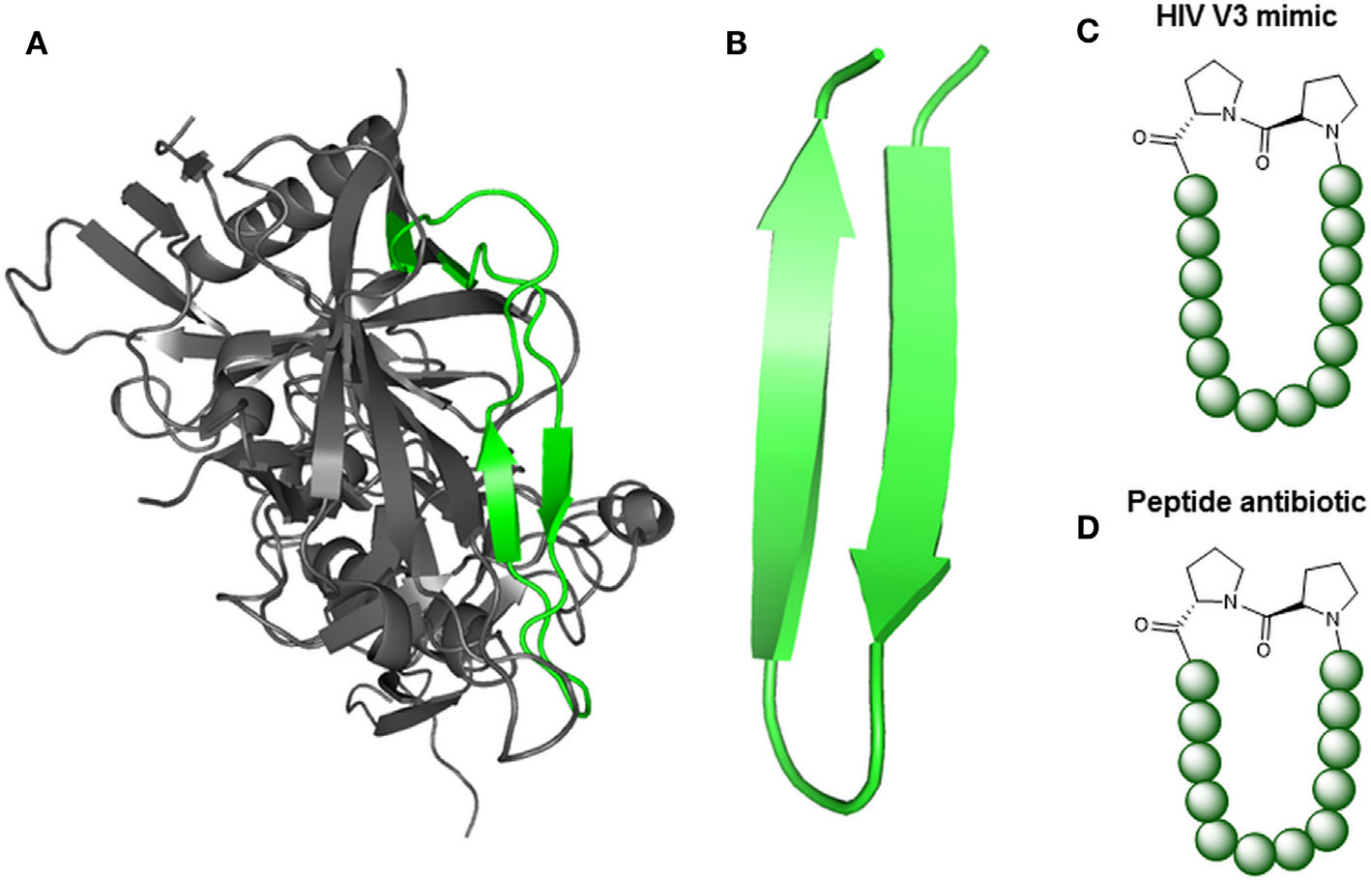
**Peptide mimics of turn structures**. **(A)** X-ray structure of gp120 [pdb 4TVP (Pancera et al., 2014)] with highlighted V3-loop (green). **(B)** NMR structure of protegrin 1 from porcine leukocytes [pdb 1PG1 (Fahrner et al., 1996)]. **(C)** V3-loop mimic, stabilized via d-Proline and l-Proline (Riedel et al., 2011).**(D)** Protegrin 1 mimic (L27-11), stabilized via d-Proline and l-Proline (Srinivas et al., 2010).

**Figure 6:**
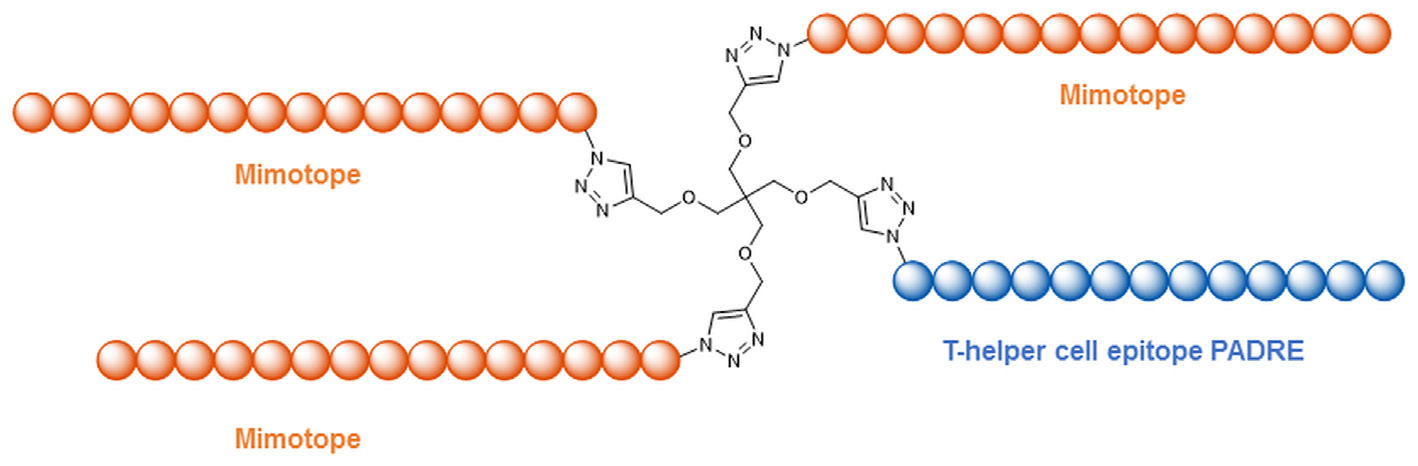
**Trimeric presentation of a b12 mimotope in conjunction with a T-helper cell epitope (Schellinger et al., 2011)**.

Understanding the molecular and structural details of the interaction of antibodies with their viral antigens is an important step in the quest for a still elusive HIV-1 vaccine (Burton et al., 2012). A prominent class of anti HIV-1 antibodies recognizes the V3-loop of the gp120 protein (Zolla-Pazner and Cardozo, 2010), which forms a β-hairpin structure when in the antibody-bound state (Figures 5A,B). Robinson et al. were able to stabilize this β-hairpin structure in V3-loop peptides by grafting them on to a d-Pro-l-Pro scaffold (Riedel et al., 2011; Robinson, 2013) (Figure 5C). Coupling of such a stabilized V3-loop mimic to a lipopeptide carrier, which self-assembles into virus-like particles (Ghasparian et al., 2011), resulted in increased immunogenicity, enabling an alternative, carrier-independent immunization. Phage display peptide libraries (Smith, 1985) have often been used to identify peptides that bind to antibodies and thus mimic their epitopes (mimotopes). Mimotopes of the broadly neutralizing HIV-1 antibody b12 have been found (Boots et al., 1997) this way. As the viral spike proteins gp120 and gp41 are presented as trimers, Schellinger et al. (2011) generated a potential immunogen based on a trimer of the b12 mimotope in conjunction with a T-helper cell epitope peptide (Figure 6). This trimeric peptide bound to b12 substantially better than the monomeric mimotope, illustrating the importance of trimeric presentation, which was achieved using the so-called click reaction (Rostovtsev et al., 2002) as a chemoselective ligation reaction.

**Figure 7:**
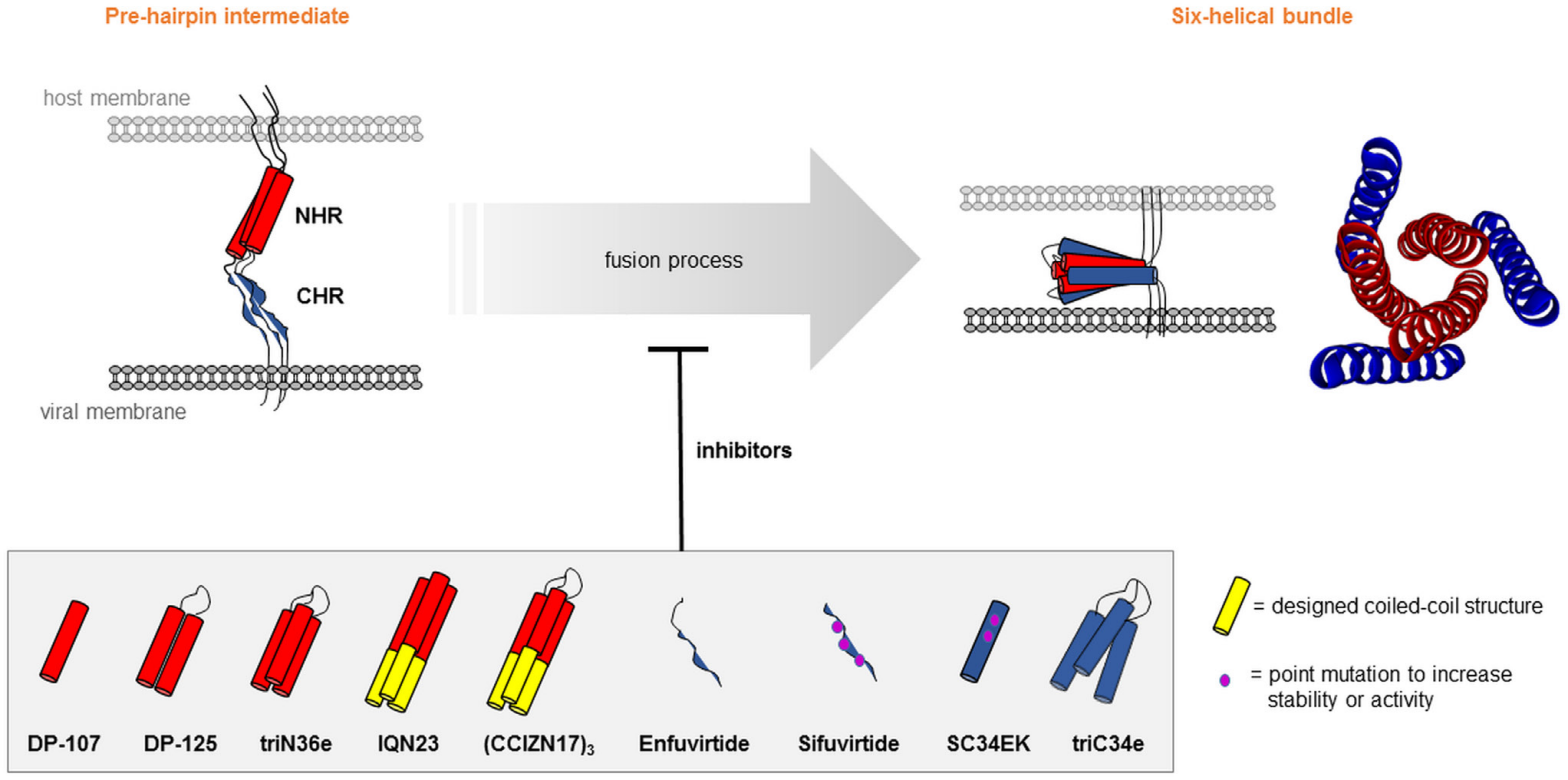
**Peptide mimics of HIV-1 gp41**. Structural rearrangements in the gp41 NHR and CHR core region during transition of the pre-hairpin intermediate to the six-helix bundle [pdb 1SZT (Tan et al., 1997)], which can be inhibited through peptides.

In addition to gp120 mimetic peptides, peptides that present parts of gp41 are also intensively researched (Cai et al., 2011). In particular this applies to peptides that mimic a six-helix bundle, consisting of a three-stranded coiled-coil structures formed by an N-terminal (NHR) and a C-terminal (CHR) heptad repeat of gp41 (Chan et al., 1997). This region of gp41 plays a key role in the process of fusion of the viral and cellular membranes (Figure 7). Peptides presenting parts of the six-helical bundle are thought to be able to interfere with its correct formation and, consequently, inhibit virus-cell fusion. Already in 1992, Wild et al. (1992) described an approach to mimic the secondary structure of NHR, which was predicted to be α-helical. Using CD spectroscopy, it could be shown that the NHR-mimetic peptide forms a stable α-helix under physiological conditions. Furthermore, the peptide exhibited a strong anti-HIV-1 activity, which could be further enhanced through dimerization. Trimers of the NHR-mimetic peptide were later found to be better HIV-1 entry inhibitors than the respective monomeric peptide (Nakahara et al., 2010). Covalent stabilization of such peptide trimers through inter-chain disulfide bridges dramatically increased the antiviral potency (Bianchi et al., 2005), as well as the HIV-1 neutralizing capacity of anti-peptide antisera (Bianchi et al., 2010).

Similar to the NHR mimics, peptides mimicking the CHR region of gp41 were developed to inhibit the formation of the six-helical bundle. In 1994, Wild et al. (1994) demonstrated a strong anti-HIV-1 activity of a peptide that overlaps the CHR. Later on, the first and so far only HIV-1 fusion inhibitor approved for clinical use (Enfuvirtide) was developed based on this peptide (Kilby and Eron, 2003; Lalezari et al., 2003). Another fusion inhibitor, called Sifuvirtide, was developed based on the 3D structure of HIV-1 gp41 and computer modeling (He et al., 2008; Wang et al., 2009). Sifuvirtide could effectively block six-helical bundle formation and was active even against Enfuvirtide-resistant HIV-1 strains. Otaka et al. increased the α-helicity of a CHR mimetic peptide by introducing Glu–Lys pairs at the *i* and *i* + 4 positions of the helix (Otaka et al., 2002), which greatly enhanced the solubility and stability of the peptide. Trimeric presentation of a CHR mimetic peptide on a C_3_-symmetric scaffold dramatically increased the antiviral activity of the peptide (Nomura et al., 2012).

### Peptides as Mimics of Cellular Receptors

Cellular receptors play important roles in signal transduction pathways, as well as in viral entry. As discussed in the previous chapter, HIV-1 contacts two receptors on the host cell surface prior to fusion with the cell membrane. Peptides that mimic these receptors are useful tools to explore the details of virus infection mechanism, as well as to develop new drugs against HIV-1. In 1998, Drakopoulou et al. (1998) developed a peptidic CD4 mimic, called CD4M, based on the analysis of site-directed mutagenesis studies, antibody-blocking experiments and the structure of the extracellular fragment of CD4, which identified the CDR H2-like loop of CD4 as the binding site for gp120 of HIV-1. To retain the native structure of the CDR H2-like loop, the peptide was transferred onto a scorpion toxin, which served as a structural scaffold. Optimizing CD4M led to a variant with100-fold increased affinity to gp120, as well as infection-inhibitory activity (Vita et al., 1999). Based on the X-ray structure of CD4 in complex with gp120 (Kwong et al., 1998), Martin et al. (2003) further optimized the CD4 mimic, resulting in a 27-mer peptide mimicking the CD4 binding site for gp120. This peptide was able to bind to gp120 at low nanomolar concentrations, inhibit binding of CD4 to gp120, as well as to induce conformational changes in gp120 similar to those triggered by CD4, from which it was derived. The importance of conformational stability of CD4 mimetic peptides could be further confirmed by Meier et al. (2012). Peptides that present the binding site of CD4 for gp120 were covalently stabilized in their loop structure by cyclization through a disulfide bond between the N- and C-terminus. Using alanine and d-phenylalanine substitution analogs, the importance of the hot spot amino acid phenylalanine 43 could be confirmed at the peptide level. These results were further confirmed by molecular dynamics simulations.

The concept of mimicking protein-binding sites through complex synthetic peptides has recently been extended to peptides that mimic the extracellular domains of seven transmembrane G protein-coupled receptors (GPCRs), which is composed of the N-terminus (NT) and the three extracellular loops (ECLs). GPCRs make up the largest class of drug targets, in fact, 27% of all clinically used drugs target a GPCR.

**Figure 8:**
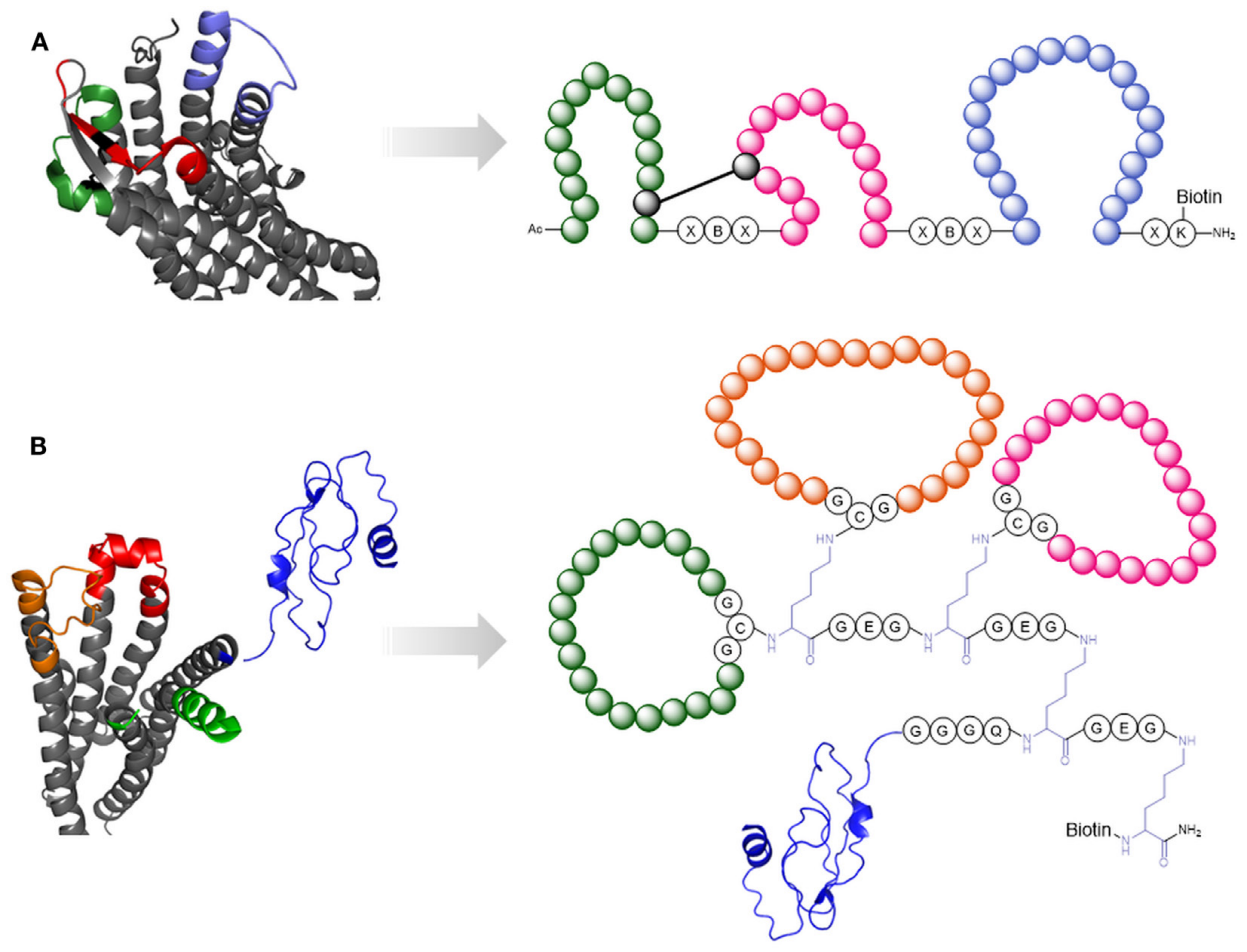
**Peptide mimics of cellular receptors**. **(A)** CXCR4 [pdb 3ODU (Wu et al., 2010)]. The extracellular loops (highlighted in green, red, and purple) are presented by the mimetic peptide CX4-M1 (Möbius et al., 2012). **(B)** GPCR CRF1 [pdb 4K5Y (Hollenstein et al., 2013)] and NMR structure of its N-terminus [pdb 2L27 (Grace et al., 2010)]. The extracellular loops are highlighted in orange, red, and green. The N-terminus is depicted in blue. These sequence stretches are presented in the CRF1 mimetic peptide (Pritz et al., 2008).

In the context of HIV-1 infection, two GPCRs are important, i.e., the chemokine coreceptors CCR5 and CXCR4. Although the 3D structures of both receptors are available (Wu et al., 2010; Tan et al., 2013), our knowledge of the structural details of their interaction with HIV-1 gp120 remains limited. Therefore, peptides that mimic the binding site of these receptors for gp120 could be useful tools for the exploration of HIV-1-coreceptor interaction at the molecular level. We have generated a peptide that mimics the three ECLs of CXCR4 (Möbius et al., 2012) (Figure 8A). This peptide, named CX4-M1, is able to discriminate between CXCR4- and CCR5-recognizing gp120 (Möbius et al., 2012) and V3-loop peptides mimicking the corresponding binding site on gp120 (Groß et al., 2013), and also inhibits HIV-1 infection of susceptible target cells in a CXCR4-specific manner (Möbius et al., 2012; Groß et al., 2015a). Furthermore, CX4-M1 is recognized by the natural CXCR4-ligand, i.e., the chemokine CXCL12 (also called SDF-1α), as well as anti-CXCR4-antibodies (Groß et al., 2015a).

In a similar approach, Pritz et al. (2008) generated, via a combination of recombinant, enzymatic and chemical synthesis, a molecule that mimics the extracellular domain of the corticotropin-releasing factor receptor type 1 (CRF1) (Figure 8B). Improving the scaffold for the presentation of the ECLs and N-terminus, as well as increasing the overall yields through synthesis optimization, enabled structural analysis of the receptor mimic – ligand interaction through NMR spectroscopy (Abel et al., 2014).

The epidermal growth factor receptor (EGFR), which is a key protein of cell proliferation and differentiation (Yarden and Sliwkowski, 2001), has also been subject to structure-based design of receptor mimetic peptides. As the receptor forms dimers or even oligomers, Hanold et al. (2015) generated a peptide mimic of the EGFR dimerization arm, which forms a β-hairpin in the native conformation. This peptide was stabilized *via* a triazole crosslink to increase proteolytic stability, while retaining the native structure, resulting in inhibition of EGFR dimerization and, consequently, a reduction of cell viability. Sequence and functional optimization of EGFR mimetic peptides may be useful for the development of novel cancer drugs addressing EGFR overexpression in tumors.

### Peptides in Cancer Research

The uncontrolled growth and spread of cells into tumor tissue (Vogelstein and Kinzler, 2004) defines cancer as one of the main fatal diseases worldwide. Therefore, a major focus in peptide drug development is on oncology (Kaspar and Reichert, 2013; Fosgerau and Hoffmann, 2015). Apart from using peptides directly as anticancer drugs (Thundimadathil, 2012), they can also serve as targeting agents to direct highly toxic chemotherapeutics to their respective targets, reducing the systemic toxicity of these drugs [for details see Kaspar and Reichert (2013)].

Structure-based approaches are often used in the design of anticancer peptides, such as the inhibitor of cell migration and invasion published by Bifulco et al. (2008). The urokinase-type plasminogen activator receptor (uPAR), which plays a critical role in cancer cell growth, survival, invasion and metastasis, contains a five amino acid sequence (SRSRY) between two of its three domains, which is exposed through ligand binding, and mediates chemotactic properties of uPAR. Using the pentapeptide SRSRY as a template, glutamine scanning and insertion of a pyroglutamine (pE) resulted in the identification of the peptide pERERY-NH_2_ as a highly active uPAR inhibitor. Further optimization through structure-based design leads to the tetrapeptide Ac-RERF-NH_2_, which is 500- to 1000-fold more active than pERERY-NH_2_ (Carriero et al., 2009). Ac-RERF-NH_2_, which has a high propensity to adopt an α-turn structure, represents a promising drug candidate against cancer.

An important target for the therapy of pancreatic, gastritic, and colorectal tumors is gastrin, a peptide hormone, whose activity can be blocked by antibodies that recognize gastrin as their epitope, delaying tumor growth (Watson et al., 1996; Barderas et al., 2008b). Detailed analysis of the antibody epitopes through alanine scanning of gastrin (Barderas et al., 2008b) and docking of the epitope into the antibody binding site, followed by affinity maturation through phage display and *in silico* methods (Barderas et al., 2008a) resulted in the development of antibody fragments with enhanced potency to inhibit gastrin-induced tumor growth. With the aim to shrink these antibodies to the size of peptidomimetics, Timmerman et al. (2005, 2009) used a strategy, in which up to three peptides derived from the complementary-determining regions (CDRs) of an antibody are presented in one molecule using the CLIPS strategy (see Toolbox for peptide synthesis: non-proteinogenic amino acids and site-selective ligation). In most cases, the activity of the obtained peptides was much lower compared to the parent antibodies. Nevertheless, neutralization of gastrin in cell-based assays by the mimetic peptides could be demonstrated (Timmerman et al., 2009). The mode of action of the peptides, however, may be different from that of the parent antibodies (Timmerman et al., 2010), leading to the conclusion that further efforts in peptide design have to be made.

Small GTPases, such as Ras, Rab, and Rho, are key proteins in many cancers, as malfunction of these proteins results in abnormal cell growth and differentiation, prolonged cell survival, membrane trafficking, and vesicular transport (Bourne et al., 1990; Cherfils and Zeghouf, 2013). Inhibiting the activity of these small GTPases could lead to new chemotherapeutic drugs for cancer treatment. One strategy to achieve this is to address the GDP–GTP exchange of Ras, which is the rate-limiting step and requires interaction with the Ras-specific guanine nucleotide exchange factor Sos (Konstantinopoulos et al., 2007). In 2011, Patgiri et al. (2011) published the structure-based design of an α-helical peptide derived from the Sos-Protein, which is able to inhibit Sos-mediated Ras activation through interference with the Sos–Ras interaction, providing a promising lead compound for anti-cancer drugs. Likewise, peptide mimics of the Rab ligands R6IP, LidA, REP1, and Rabin8 have been reported (Spiegel et al., 2014). Using the hydrocarbon-peptide stapling approach, α-helical peptides were stabilized at positions *i* and *i* + 4, resulting in up to 200-fold increased affinity of the peptide to Rab proteins. In addition, one of the peptides, being a pioneer inhibitory compound for Rab GTPase–protein interactions, was found to inhibit the Rab8a–effector interaction.

A challenge in cancer drug delivery is the discrimination between self and non-self, i.e., clearance of drug-loaded nanoparticles before they reach their target. To overcome this problem, synthetic polymers such as polyethylene glycol are used, but these can hamper uptake by cancer cells (Hong et al., 1999a). As an alternative strategy, Rodriguez et al. (2013) generated, based on the crystal structure of the hCD47–hSIRPα complex, and in combination with computational simulations, a minimal “self” 21-mer peptide. This peptide, which originates from CD47, an established marker of “self” (Rodriguez et al., 2013), was able to prolong the circulation of nanobeads in mice by preventing phagocytosis, providing a new opportunity for enhanced delivery of drugs or imaging agents. As an example for its utility as a marker of “self,” the anti-cancer drug paclitaxel was loaded onto nanoparticles, which also presented the marker-peptide on their surface. Due to delayed clearance, treatment with peptide-coated nanoparticles induced a more efficient size-reduction of lung adenocarcinoma epithelial tumors in mice than beads without the peptide. Although this peptide is not the bio-active compound, it provides an excellent tool for the delivery of drugs to tumor tissues.

Peptides are also promising candidates for cancer immunotherapy, where they are used as vaccines that present tumor-associated antigens, which trigger an immune response against the tumor in the patient. It can be expected that peptides presenting tumor-associated antigens will increasingly gain significance for cancer immunotherapy in the future (Miller et al., 2013).

### Peptides as Antibiotics and Anti-inflammatory Compounds

The growing multi-resistance of bacteria to clinically used antibiotics is one of the current challenges in biomedical research (Dennesen et al., 1998). The development of new antibacterial drugs is therefore an urgent necessity, and peptides have proven beneficial in this area of drug development as well. Robinson et al. (2005) could demonstrate improved antimicrobial activity, as well as plasma half-life of β-hairpin mimics of the naturally occurring membranolytic host-defense peptide protegrin 1 (Shankaramma et al., 2002; Srinivas et al., 2010) (Figure 5D). These peptides were cyclized via a d-proline–l-proline template, reducing flexibility and stabilizing the conformation of the peptide (Shankaramma et al., 2002; Robinson et al., 2005; Srinivas et al., 2010). Furthermore, these peptides were shown to directly interact with the bacterial β-barrel protein LptD, which sets them apart from other antimicrobial peptides, whose effect is mainly based on a membranolytic activity.

**Figure 9:**
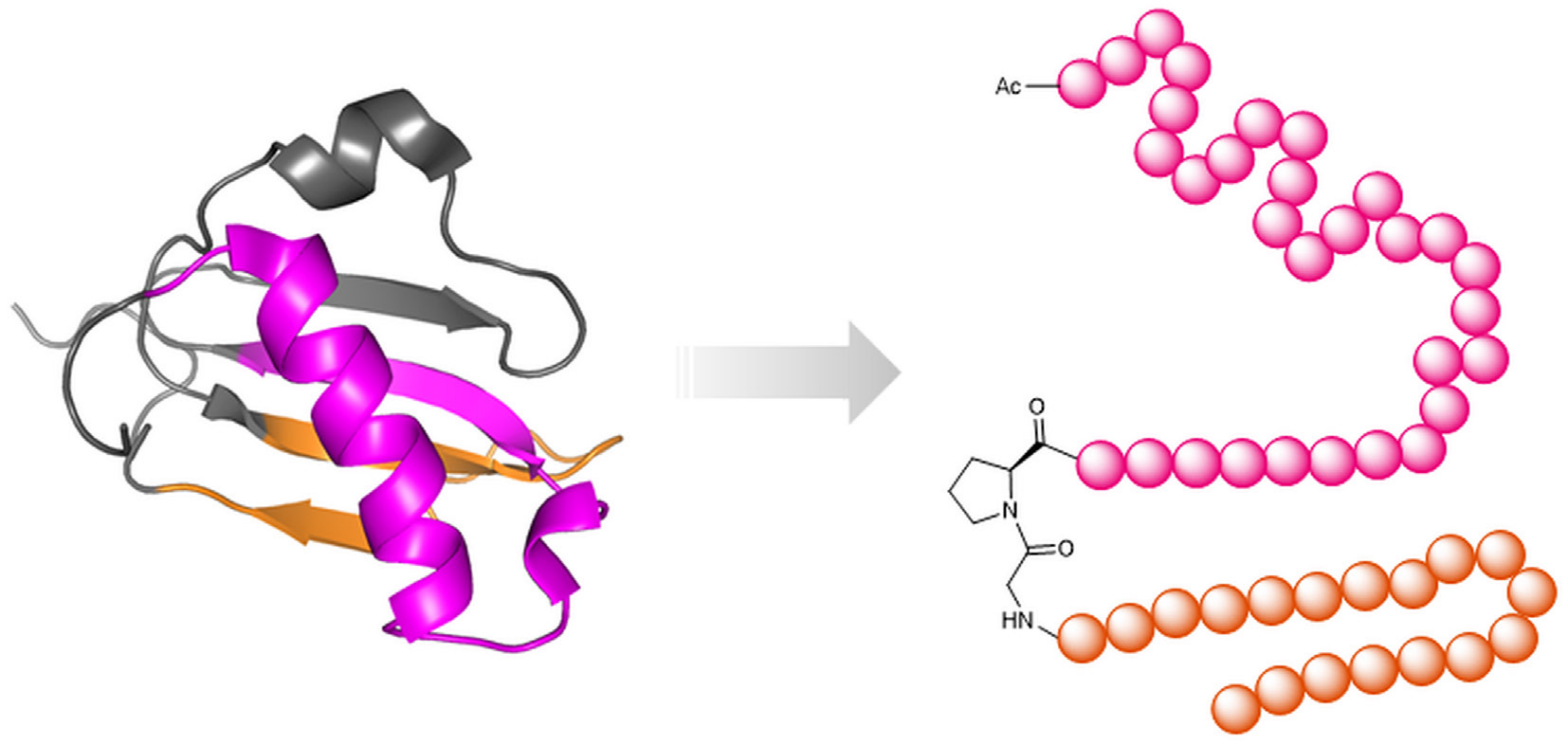
**Peptide mimics of an anti-inflammatory protein**. Left: NMR structure of the chemotaxis inhibitory protein of *S. aureus* [CHIPS31-121 pdb 1XEE (Haas et al., 2005)] with highlighted discontinuous binding site for the C5a receptor, which was mimicked through the peptide CHOPS (Bunschoten et al., 2011) (right).

An anti-inflammatory peptide, named CHOPS (Bunschoten et al., 2011) (Figure 9), was designed based on the structure of the chemotaxis inhibitory protein of *Staphylococcus aureus* (CHIPS) (Veldkamp et al., 2000; Haas et al., 2005; Ippel et al., 2009). CHIPS is known to bind to the C5a-receptor and to inhibit the C5a–C5a-receptor interaction (Postma et al., 2004), thus addressing an important element in the complement cascade of the innate immunity. As full-length CHIPS is highly immunogenic (Gustafsson et al., 2009), its peptide mimic CHOPS, whose conformation is similar to the respective CHIPS fragment, and which binds to the N-terminus of the C5a-receptor (Bunschoten et al., 2011), may become a promising alternative for the treatment of inflammatory and autoimmune diseases.

Proteins in the outer membrane of Gram-negative bacteria often have β-barrel structures. The proper assembly of these proteins is provided for by the β-barrel assembly machine (Bam) (Hagan et al., 2011). One important component of the Bam multiprotein complex is BamD, which interacts with unfolded protein substrates, like BamA, and facilitates their assembly in the outer membrane (Hagan et al., 2013). Using a peptide scanning approach of the C-terminal region of BamA, a 15-mer peptide was identified as an inhibitor of outer membrane protein assembly (Hagan et al., 2015). *In vivo* expression of this peptide resulted in bacterial growth defects, and sensitized resistant *Escherichia coli* to antibiotics, marking a starting point for the development of new antibiotic compounds for gram-negative bacteria (Hagan et al., 2015).

It should also be noted that a plethora of antimicrobial peptides are found in numerous organisms, including insects, mammals, plants, and bacteria (Mojsoska et al., 2015), which are not subject of this review. Furthermore, computer-based design strategies are aimed at the design of antimicrobial peptides with improved activity and reduced mammalian cell toxicity (Fjell et al., 2012).

## Challenges and Future Directions

Due to their intrinsic properties, such as their potential for highly specific interactions with target molecules, generally low toxicity and immunogenicity, and rapid clearance, peptides are increasingly appreciated as candidates for novel drugs. This is particularly true for the development of protein–protein interaction inhibitors, where peptides are often better able than small molecules to cover large protein interface areas.

On the other hand, peptides also present severe bottlenecks that need to be considered and, if necessary, addressed in the development of peptide drugs. The biggest challenge clearly is the limited metabolic stability of peptides, since they are rapidly degraded by proteolytic enzymes, precluding oral administration of peptide drugs. This challenge can be addressed by different means. First, unlike recombinant protein synthesis, chemical peptide synthesis is not limited to the proteinogenic amino acids as building blocks. A plethora of additional amino acids are currently available for chemical peptide synthesis. Apart from dramatically increasing the metabolic stability of peptides, incorporation of these amino acids also increases the chemical diversity presented by synthetic peptides, as these additional amino acids introduce chemical moieties that are not presented by the proteinogenic amino acids. Furthermore, conformational stabilization through cyclization, or through introduction of defined secondary structures, has been shown to shield peptides from proteolytic enzymes. Such shielding effects can also be achieved by coupling the peptide to larger inert molecules, such as polyethylene glycol (Swierczewska et al., 2015).

Due to their molecular size, peptides are rarely able to passively pass cell membranes, limiting their utility to address intracellular target molecules. This drawback, however can be counteracted by attaching the drug peptide to one of a large group of available cell-penetrating peptides (Kurrikof et al., 2015), which are able to transport a variety of molecular cargo into cells.

In general, the chemical synthesis of peptides through solid-phase synthesis is fairly straightforward and has been optimized over the past decades, so that virtually all peptide sequences are accessible synthetically today. In our experience, however, the synthesis of specific peptides may require the use of specific protected amino acids and other building blocks, solid supports, linkers and other reagents, which significantly increases the cost of synthesis. These considerations may become relevant for the large-scale synthesis of peptide drugs, as well as peptide biomaterials.

The design of peptides as protein–protein interaction inhibitors is typically based on the resolved 3D structure of the respective protein–protein complex. While such structures are increasingly becoming available through powerful x-ray crystallography technology, their generation is not trivial and contingent on the availability of suitable crystals of the protein complexes.

Overall, taking into account the tremendous technical and scientific progress in the field of using peptides as protein mimics, we strongly believe that the significance of synthetic peptides in biomedical research, as well as in biomaterial engineering, will continue to grow in the future.

## Conclusion

The design of peptides as protein mimics has evolved as a promising strategy for the exploration of, as well as the controlled interference with, protein–protein interactions. Due to their chemical nature, peptides are an appropriate type of molecules for the mimicry of protein-binding sites, including those involving large protein–protein interfaces. The possibility to use non-proteinogenic amino acids, as well as various methods of chemical modification, greatly enhances the scope of chemical and structural versatility, as well as stability, of synthetic peptides. Apart from their significance as molecular tools to explore protein–protein interactions, such protein mimetic peptides are also candidates for the inhibition of protein–protein interactions involved in disease processes. Furthermore, peptides play an important role in biomaterial engineering, as they are biocompatible, biodegradable, and functionally selective. Photo-switchable peptides can be used to temporally and/or spatially control processes in organisms, such as drug release at specific organs or tissues. These applications illustrate the utility and versatility of synthetic peptides as molecular tools in biomedical research, as well as in synthetic biology.

## Author Contributions

AG and CH have written individual chapters and prepared figures. HS and JE have written and edited the manuscript.

## Conflict of Interest Statement

The authors declare that the research was conducted in the absence of any commercial or financial relationships that could be construed as a potential conflict of interest.
